# Costs, Reach, and Benefits of COVID-19 Pandemic Electronic Benefit Transfer and Grab-and-Go School Meals for Ensuring Youths’ Access to Food During School Closures

**DOI:** 10.1001/jamanetworkopen.2022.29514

**Published:** 2022-08-31

**Authors:** Erica L. Kenney, Lina Pinero Walkinshaw, Ye Shen, Sheila E. Fleischhacker, Jessica Jones-Smith, Sara N. Bleich, James W. Krieger

**Affiliations:** 1 Department of Nutrition, Harvard T.H. Chan School of Public Health, Boston, Massachusetts; 2 Department of Health Systems and Population Health, University of Washington, Seattle; 3 Center for Health Decision Science, Harvard T.H. Chan School of Public Health, Boston, Massachusetts; 4 Georgetown University Law Center, Washington, DC; 5 Department of Epidemiology, University of Washington, Seattle; 6 Department of Health Policy and Management, Harvard T.H. Chan School of Public Health, Boston, Massachusetts

## Abstract

**Importance:**

School meals are associated with improved nutrition and health for millions of US children, but school closures due to the COVID-19 pandemic disrupted children’s access to school meals. Two policy approaches, the Pandemic Electronic Benefit Transfer (P-EBT) program, which provided the cash value of missed meals directly to families on debit-like cards to use for making food purchases, and the grab-and-go meals program, which offered prepared meals from school kitchens at community distribution points, were activated to replace missed meals for children from low-income families; however, the extent to which these programs reached those who needed them and the programs’ costs were unknown.

**Objective:**

To assess the proportion of eligible youths who were reached by P-EBT and grab-and-go meals, the amount of meals or benefits received, and the cost to implement each program.

**Design, Setting, and Participants:**

This cross-sectional study was conducted from March to June 2020. The study population was all US youths younger than 19 years, including US youths aged 6 to 18 years who were eligible to receive free or reduced-price meals (primary analysis sample).

**Exposures:**

Receipt of P-EBT or grab-and-go school meals.

**Main Outcomes and Measures:**

The main outcomes were the percentage of youths reached by P-EBT and grab-and-go school meals, mean benefit received per recipient, and mean cost, including implementation costs and time costs to families per meal distributed.

**Results:**

Among 30 million youths eligible for free or reduced-price meals, grab-and-go meals reached an estimated 8.0 million (27%) and P-EBT reached 26.9 million (89%). The grab-and-go school meals program distributed 429 million meals per month in spring 2020, and the P-EBT program distributed $3.2 billion in monthly cash benefits, equivalent to 1.1 billion meals. Among those receiving benefits, the mean monthly benefit was larger for grab-and-go school meals ($148; range across states, $44-$176) compared with P-EBT ($110; range across states, $55-$114). Costs per meal delivered were lower for P-EBT ($6.46; range across states, $6.41-$6.79) compared with grab-and-go school meals ($8.07; range across states, $2.97-$15.27). The P-EBT program had lower public sector implementation costs but higher uncompensated time costs to families (eg, preparation time for meals) compared with grab-and-go school meals.

**Conclusions and Relevance:**

In this economic evaluation, both the P-EBT and grab-and-go school meal programs supported youths’ access to food in complementary ways when US schools were closed during the COVID-19 pandemic from March to June 2020.

## Key Points

**Question:**

What were the operating costs, costs and benefits to families, and proportion of eligible youths who received benefits of 2 programs aimed at replacing school meals missed when US schools were closed owing to COVID-19 from March to June 2020?

**Findings:**

In this economic evaluation, among 30 million youths eligible to receive free or reduced-price meals, the Pandemic Electronic Benefit Transfer (P-EBT) program (state agencies sent debit cards loaded with the cash value of missed school meals to families) reached 89% of eligible students and cost $6.46 per meal. Grab-and-go school meals (school food service departments provided prepared meals for off-site consumption) reached 27% and cost $8.07 per meal.

**Meaning:**

These findings suggest that during times when youths cannot access school meals, state and federal agencies should support cost-efficient programs for schools to distribute prepared meals and activate programs such as P-EBT to efficiently reach eligible youths.

## Teaser

This economic evaluation evaluates the reach, costs, and benefits of the Pandemic Electronic Benefit Transfer and grab-and-go meals programs for students during COVID-19–related school closures in the US from March to June 2020.

## Introduction

Ensuring that children have adequate nutrition (continuous access to enough healthy food to promote healthy development) is a critical public health challenge.^1,2,3^ Inequitable access to healthy food is associated with racial and socioeconomic disparities in diet quality from early ages.^4,5,6^ Children from low-income households are at increased risk for experiencing food insecurity and inadequate nutrition^7^ and have a disproportionate burden of childhood obesity^8,9^; thus, they are at higher risk of experiencing multiple nutrition-related threats to their health.^10^

The National School Lunch and Breakfast Programs administered by the US Department of Agriculture (USDA) have been found to have an important influence on children’s nutrition. They are associated with significantly reduced food insecurity,^11,12^ improved diet quality, and reduced obesity risk among children from low-income households.^13,14,15^ Before the COVID-19 pandemic, more than 30 million children received National School Lunch Program meals each year, 22 million of whom were from households with incomes of 185% or less of the federal poverty level, thus qualifying them for free or reduced-price meals (FRPM).^16^

However, COVID-19–related school closures in spring 2020 disrupted access to school meals for millions of US children, increasing the risk of food insecurity for children depending on this food source. In response, US Congress authorized the USDA to implement 2 approaches: (1) grab-and-go school meals, in which school food authorities switched from preparing meals for students to eat inside schools to distributing prepared meals for off-site consumption through community distribution sites or mobile delivery systems, and (2) the Pandemic Electronic Benefit Transfer (P-EBT) program, in which states distributed the cash value of missed school meals to parents of FRPM-eligible children on a debit-like card so they could purchase groceries from food retailers, similar to EBT cards used in the Supplemental Nutrition Assistance Program. Preliminary reports suggest that both programs helped alleviate household food insecurity.^17,18^ However, it is not clear how effective the programs were at distributing benefits to eligible children during school closures or how much the programs cost.

This study’s aim was to estimate the proportion of FRPM-eligible youths reached, program implementation and family costs, benefits received by participating youths, and cost per meal distributed for the 2 programs during the spring of 2020, a period when nearly all schools across the US were closed.^19^

## Methods

### Study Design and Population

This economic evaluation followed the Consolidated Health Economic Evaluation Reporting Standards (CHEERS) reporting guideline^20^ and used an existing analytic framework^21^ to evaluate the costs, population reach, and benefits distributed of P-EBT and grab-and-go school meals. Our primary analysis examined the extent to which these programs reached youths aged 6 to 18 years who were living in the US and were eligible for FRPM because this was the population most affected by school closures. This population included youths who were eligible because their families had incomes of 185% or less of the federal poverty level and those attending school districts that participated in the Community Eligibility Program (in which districts with large proportions of children from low-income households offer free meals to all children regardless of income). Because schools provided grab-and-go meals to all children younger than 19 years (regardless of income or Community Eligibility Program participation), we also evaluated how extensively this meal program reached this broader population. We leveraged multiple government and administrative data sets with national- and state-level data on program participation, costs, and benefits from March to June 2020 (Table 1).^22,23,24,26,27,28,29^ Analyses included all states except for South Dakota and Wyoming, which were excluded owing to insufficient data. Review and approval of this study was not required per the guidelines of the University of Washington institutional review board because it was non–human participant research.

**Table 1.** Data Sources for Estimating Program Reach, Benefits, and Implementation Costs for Grab-and-Go School Meals and the P-EBT Programs in Spring 2020

Source	Data description
**Reach**	
Household Pulse Survey^22^	No. and proportion of FRPM-eligible families who reported picking up a free school meal
P-EBT 2019-2020 SY state participation^23^	Total No. of P-EBT/FRPM-eligible children per state (for some states)
American Community Survey^24^	No. of children aged 0-19 y per state
P-EBT Implementation Documentation Project^25^	Surveys of total No. of P-EBT/FRPM-eligible children; No. of times P-EBT benefits were issued in each state
State government websites and press releases	No. of children receiving P-EBT
P-EBT distribution data^26^	No. of children receiving P-EBT
**Benefits**	
Child nutrition tables	Total meals distributed in each state for NSLP, NSBP, SSO, and SFSP in April and May 2020
P-EBT distribution data^26^	Total dollar amounts disbursed per state per month from March to June 2020
P-EBT 2019-2020 SY state participation^23^	Planned or budgeted dollar amounts to be disbursed for P-EBT from March to June 2020
**Costs**	
P-EBT 2019-2020 SY state participation^23^	State-requested P-EBT administrative funding for the 2020-2021 academic school year
Kenney et al,^27^ 2021	District-level cost associated with delivering school meals (including administrative, operating, and food costs) for 7 of the largest US school food authorities
School meal cost survey (primary data)	District-level costs associated with delivering school meals (including administrative, operating, and food costs) for a convenience sample of 17 districts nationally
Davis and You,^28^ 2010	Estimated cost to FRPM-eligible family (including time and wage) to prepare home meals
Voulgaris et al,^29^ 2017	Estimated travel cost for a family to reach a school site to pick up a school meal to go (including time and mileage costs, taking into account drivers and bus riders)

Abbreviations: FRPM, free or reduced-price meal; NSBP, National School Breakfast Program; NSLP, National School Lunch Program; P-EBT, Pandemic Electronic Benefit Transfer; SFSP, Summer Food Service Program; SSO, Seamless Summer Option; SY, school year.

### Program Reach

Program reach was defined as the proportion of youths eligible for each program who received benefits regardless of whether they actually used them (ie, consumed the meals or spent the P-EBT benefits). The eligible population for P-EBT was FRPM-eligible students. The population eligible for grab-and-go meals was all youths younger than 19 years; we also focused on the subset of this population that consisted of FRPM-eligible students. The number of FRPM-eligible students in each state was obtained from state applications to the USDA for P-EBT funding combined with survey data.^23,25^ The number of all youths younger than 19 years was obtained from the American Community Survey.^24^

To estimate how many youths nationally and in each state benefitted from grab-and-go school meals in spring of 2020, we used Household Pulse Survey data from the US Census Bureau,^22^ which collected weekly nationally representative data on household composition, income, and whether or not a household had received free meals from schools. We calculated national and state-stratified estimates of the proportions of households with youths younger than 19 years and households eligible for FRPM that reported receiving free meals from schools. To estimate the number of youths who received P-EBT, we triangulated data from 3 sources. First, if available, we used estimates from state government websites (eg, press releases) or data released through a Freedom of Information Act request (available from 22 states). Next, in states where this was not available, we used states’ estimates to the USDA of the amount of cash they planned to disburse for spring 2020 and the total number of days that benefits were issued.^23^ Lastly, in states where their initial plans for distribution were inaccurate, we used USDA-released data on the number of households receiving benefits in a given month.^26^

### Program Benefit*s*

Program benefits for participating youths were estimated as the monthly cash value of the benefits and the number of meals or meal equivalents received. The number of meals provided nationally and per state by grab-and-go school meals programs during April and May 2020 by FRPM eligibility was obtained from the USDA. The cash value of benefits provided per state for P-EBT was estimated using data from states’ P-EBT applications to the USDA^23^ and administrative data from the USDA on benefit disbursal from March through June 2020.^26^ We converted the meals distributed through the grab-and-go meals program to their cash value using the USDA reimbursement value for 2020 ($5.85 for 2 meals per day in the continental US). Similarly, we converted P-EBT cash benefits to meal equivalents by dividing the total cash benefits distributed by the cash benefit distributed per day ($5.70 in the continental US) and then multiplying by 2 because 2 meals per day were meant to be covered. If, owing to some measurement error in the administrative data, the estimate for the mean number of meals distributed per youth per month for grab-and-go meals for a state was greater than 60 (the maximum possible number given 2 meals per a 30-day month), we capped this measure at 60 meals per month (for 14 states). Similarly, for P-EBT, if a state estimate was greater than 40 meals (the maximum possible meal equivalents), we capped the value at 40 (for 28 states).

### Cost per Meal Delivered

#### Costs of Grab-and-Go Meals

We followed standard guidelines for identifying, measuring, and valuing the resources contributing program costs.^30,31,32^ We combined data from an existing study of school food costs^27^ with data we collected from a survey of a convenience sample of school food service directors from 17 districts, resulting in a total sample of 24 school districts across 9 states. The sample included states from every US census region and districts of varying size, with the number of daily lunches served ranging from 2400 to 3 140 000. Data included labor (including fringe benefits), food (including food waste), and operating costs (eg, personal protective equipment, transportation, and take-out containers) associated with grab-and-go meal implementation. Surveys also captured the number of meals served in each district, allowing us to standardize costs per meal. We additionally accounted for uncompensated costs to families, namely, the mean time spent and travel costs expended for families to pick up meals, using previously published estimates of the travel time for students to their local schools^29^ and the value of adult caregivers’ time in FRPM-eligible households.^28^ These costs were then weighted by district size and by the number of FRPM-eligible students in sampled states to estimate weighted mean state- and national-level costs.

#### Costs of P-EBT

To estimate the costs of P-EBT, we used each state’s P-EBT application to the USDA,^23^ which included estimates of state-specific labor costs for identifying and contacting eligible participants, distributing benefits, and monitoring the program, as well as the cash value of the P-EBT benefits disbursed and equipment costs. We then summed costs across all states to calculate national program costs. For uncompensated costs to families, we accounted for caregivers’ time spent preparing meals with foods purchased through P-EBT using existing estimates of preparation time for breakfasts and lunches in low-income households^33^ and the aforementioned valuation of FRPM-eligible caregivers’ time.^28^

#### Cost per Meal

To calculate the cost per meal delivered for each program from a societal perspective, we divided the national monthly cost of meals distributed by the national monthly number of meals or meal equivalents delivered per month. In a secondary analysis, we calculated the cost per meal provided for public agencies and families.

### Statistical Analysis

The analyses described above were conducted using Microsoft Excel and StataMP, version 14 (StataCorp LLC).^34^ More details on methods and calculations are given in eAppendixes 1 to 5 in the Supplement.

## Results

During April and May 2020, an estimated 8 million of the 30 million FRPM-eligible youths (27%) in the US were reached by the grab-and-go school meals program. Among all 69.8 million youths aged 2 to 18 years, grab-and-go school meals reached an estimated 10.5 million (15%). The estimated reach of grab-and-go meals for FRPM-eligible students varied substantially by state, ranging from 14% to 54% (Table 2 and Figure). Meanwhile, P-EBT reached an estimated 26.9 million FRPM-eligible children (89%), with the percentage reached also varying substantially across states, from 51% to 100%.

**Table 2.** National Reach, Benefits, and Implementation Costs per Month for Grab-and-Go School Meals and the P-EBT Programs From March to June 2020^a^

	Grab-and-go school meals		P-EBT	
	Weighted national mean	Range across states	Weighted national mean	Range across states
FRPM-eligible students reached, No. (%)	8.0 Million (27)	14-54	26.9 Million (89)	51-100
Youths aged 2-18 y reached, No. (%)	10.5 Million (15)	7-30	NA	NA
Monthly program benefit per FRPM-eligible recipient, $	148	44-176	110	55-114
Monthly program benefit per FRPM-eligible recipient, meals/meal equivalents	50	15-60	39	18-40
Cost per meal delivered per FRPM-eligible recipient, $	8.07	2.97-15.27	6.46	6.41-6.79

Abbreviations: FRPM, free or reduced-price meal; NA, not applicable; P-EBT, Pandemic Electronic Benefit Transfer.

^a^ South Dakota and Wyoming were excluded from analyses owing to insufficient data.

**Figure.**
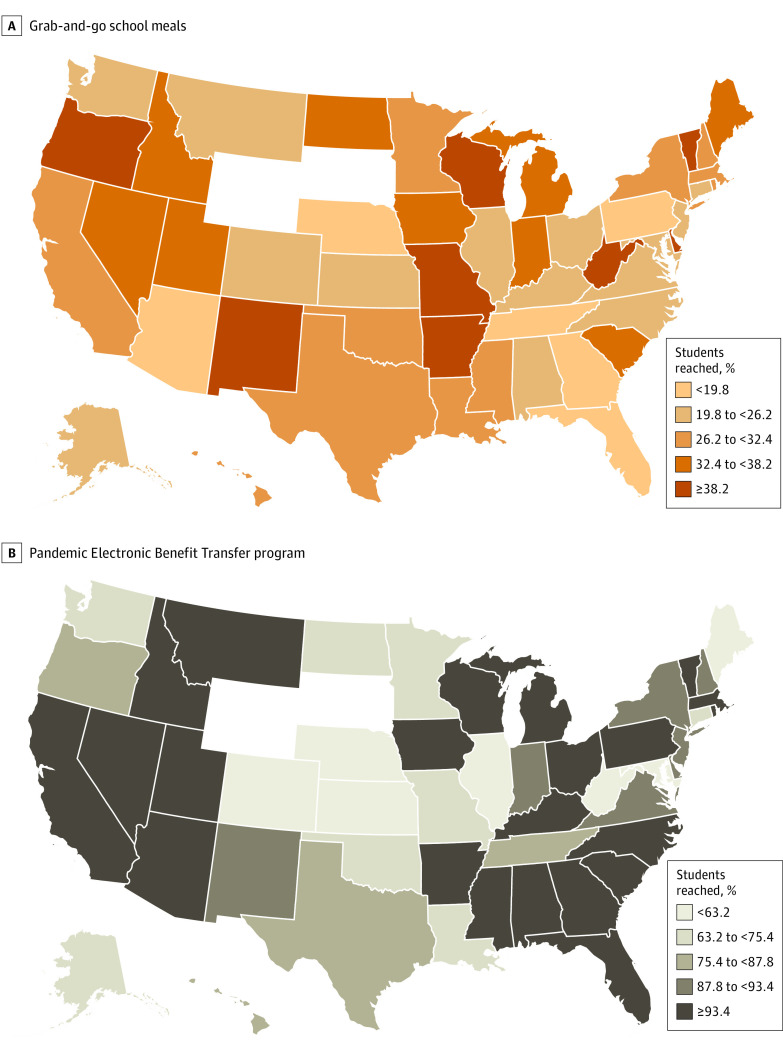
Percentage of Students Eligible for Free or Reduced-Price Meals Who Participated in Grab-and-Go School Meals and Pandemic Electronic Benefit Transfer Programs by State in Spring 2020

The grab-and-go school meals program distributed a mean of 429 million meals per month, with an estimated value of $1.2 billion, in spring 2020. This translated to an estimated mean of 50 meals distributed per month per FRPM-eligible youth (range across states, 15-60 meals), with a retail cash value of approximately $148 per month per youth (range across states, $44-$176).

States issued a mean of $3.2 billion in monthly cash benefits, equivalent to 1.1 billion meals, through P-EBT in from March to June 2020. However, not all these benefits were actually received by families during this period; in many states, families did not receive benefits until August or September 2020 owing to P-EBT implementation delays. This translated to an estimated $110 in cash-value benefits per month per youth receiving P-EBT benefits (range across states, $55-$114), or approximately 39 meals that could potentially have been purchased with P-EBT benefits per month per youth (range across states, 18-40 meals).

The total national mean weighted cost per meal provided was $8.07 (range across states, $2.97-$15.27) for grab-and-go school meals and $6.46 (range across states, $6.41-$6.79) for P-EBT. The uncompensated cost for families for grab-and-go meals ($1.00 per meal) was lower than for P-EBT ($3.56 per meal). The public agency cost component was lower for P-EBT ($2.90 per meal) compared with grab-and-go school meals ($7.07 per meal).

## Discussion

School meal programs have an essential role in ensuring that millions of US children, particularly those living in households near or in poverty, have access to meals that promote healthy growth.^35^ For most US children, a school meal is the most nutritious meal of their day.^36^ The best approaches to ensuring access to nutritious meals when schools are closed, whether during emergencies or routine closures such as summer vacation, are thus important policy issues. This study suggests that, in many states, P-EBT can reach FRPM-eligible youths at relatively low cost to the government, while a meal distribution model such as grab-and-go school meals can also ensure that families directly receive meals and reach youths beyond those who are FRPM eligible. Our results and those of other researchers suggest that disaster preparedness plans that help school meal programs safely set up community distribution sites^27,37,38,39,40^ and establish infrastructure so that P-EBT can be rapidly deployed^40^ are needed to prevent future disruption of food access for children. Our P-EBT findings also suggest that there may be a benefit to scaling up USDA’s Summer EBT program, which is currently a pilot program.^41^

Although P-EBT was implemented at varying paces across the states,^25^ with many families not receiving the benefits allotted to them for spring 2020 until later in the year, our analysis suggests that during a time of uniform school closures, P-EBT reached most FRPM-eligible youths nationally. The P-EBT program provided benefits to 89% of FRPM-eligible youths compared with 27% of youths reached by grab-and-go school meals. Grab-and-go school meals may have reached fewer families because families typically had to travel to a distribution site at fixed times to obtain meals. Travel was often a challenge^40^ despite substantial efforts by school food authorities to make meal acquisition feasible.^27,38^ In contrast, the effort required for households to receive P-EBT was relatively low because cards were automatically mailed to families of youths known to be eligible and could be used to buy food at any store accepting EBT.

In this study, the per-meal cost of P-EBT ($6.46) was less than that of grab-and-go school meals ($8.07). The lower costs of P-EBT may be related to the fewer resources required for implementation. In contrast, grab-and-go school meals required more resources, particularly labor and equipment, owing to the need to source, prepare, package, distribute, and seek reimbursement for meals.^42^ Prior research has shown that delivery of grab-and-go school meals is more costly compared with providing regular onsite school meals when schools are open.^27^

Although our analyses suggest that P-EBT reached more FRPM-eligible youths at a lower cost per meal, P-EBT should not be the sole response to address youths’ meal access when schools are closed. Given the initial delays in implementation of P-EBT and potential difficulties in implementing the program when schools are not uniformly closed, P-EBT may not in its current configuration quickly reach all students. The program may need reconfiguration to simplify determination of missed meals and streamline benefit distribution.

Grab-and-go school meals were also an important safety net for families cut off from school meals but not able to access P-EBT, including the approximately 3.5 million households with food-insecure youths that were not income eligible for FRPM,^7^ families that experienced sudden loss of income owing to the pandemic that made them eligible for P-EBT but who might not have been captured by administrative data as being eligible, and those without stable housing (eg, 2.7% of students experience homelessness^43^) or accurate mailing addresses to allow delivery of P-EBT cards. Grab-and-go school meals also may be associated with reduced time cost of preparing meals for children, which can be high for low-income caregivers.^28^ School employees have noted that grab-and-go school meals programs allowed school staff to maintain contact with families who would otherwise have been isolated and to distribute home-learning resources.^27^ Lastly, the grab-and-go school meals had to meet at least basic USDA nutritional standards, whereas P-EBT could be used to purchase any type of food. Thus, although P-EBT gave parents more choice, the foods purchased with P-EBT benefits may not have been as nutritionally sound as grab-and-go school meals.

School closures in response to the COVID-19 pandemic created a challenge for children and families who depend on school meals. The combination of P-EBT and grab-and-go school meals offers a 2-pronged strategy to facilitate food access when schools are closed during emergencies or routine breaks. The ability of P-EBT to reach nearly all eligible students from low-income households may prevent gaps in food access owing to school closures, and grab-and-go school meals could reach households that are not eligible for P-EBT benefits and provide meals that meet USDA nutrition standards. This study suggests that future development of the programs should consider (1) investigating strategies for cost containment for grab-and-go school meals; (2) expanding P-EBT to cover 60 meals per month instead of 40 to match the grab-and-go school meals benefit level; (3) exploring the use of P-EBT during all times when schools are closed for lengthy periods (eg, summer vacations), not just emergencies; and (4) optimizing the nutritional quality of the foods provided.

### Limitations

This study has limitations. First, P-EBT administrative data from some states did not separate distribution of P-EBT and Supplemental Nutrition Assistance Program benefits, which made it impossible to accurately estimate the number of unique P-EBT recipients and the benefits they received using a single data source for those states; thus, it was necessary to triangulate several data sources. In some states, estimates of benefits provided by both P-EBT and grab-and-go meals exceeded the maximum permitted by the USDA, leading us to cap estimated benefits distributed per person if they exceeded this threshold. High estimates may have resulted from the previously mentioned limitations of P-EBT data, errors in states’ reports of distributed grab-and-go meals, or errors in estimates of the number of FRPM-eligible students. Household Pulse Survey data,^22^ which we used to estimate the number of youths receiving grab-and-go school meals, assessed receipt at the household, not child, level. In addition, we were unable to locate a source of data for federal program administrative costs (ie, at the USDA). However, these costs were likely similar for the 2 programs and small compared with the larger cost contributions attributable to the benefits and labor costs.

Our study examined the start-up period of both programs. Additional analysis could assess program reach when school closures are more inconsistent and whether costs might decrease once programs are mature. The range in estimated costs across states suggests that some states may have developed cost-efficient approaches for program implementation. Identifying these could inform future program implementation.

In addition, we were unable to identify data to assess the association of the programs with food and nutrition security among youths. It would be useful to understand the extent to which P-EBT benefits were redeemed, whether they were used to buy food for household youths, and the nutritional quality of the foods purchased with them. Similarly, future research should evaluate the degree to which grab-and-go school meals were consumed, who consumed them, and their effects on diet quality.

## Conclusions

In this economic evaluation, both the P-EBT and grab-and-go school meal programs supported youths’ access to food in complementary ways when US schools were closed during the COVID-19 pandemic from March to June 2020. These findings suggest that during times when youths cannot access school meals, state and federal agencies should support cost-efficient programs for schools to distribute prepared meals and activate programs such as P-EBT to efficiently reach eligible youths.

## Notes

Supplement. **eAppendix 1.** Formulas for Calculating Metrics **eAppendix 2.** Methods for Estimating Proportion of Families Collecting Grab-and-Go Meals **eAppendix 3.** Decision Process for Selecting Sources for P-EBT Reach and Benefits **eAppendix 4.** Calculation of Grab-and-Go School Meals Costs **eAppendix 5.** Calculation of P-EBT Costs
